# The Role of Wild-Type RAS in Oncogenic RAS Transformation

**DOI:** 10.3390/genes12050662

**Published:** 2021-04-28

**Authors:** Erin Sheffels, Robert L. Kortum

**Affiliations:** Department of Pharmacology and Molecular Therapeutics, Uniformed Services University of the Health Sciences, Bethesda, MD 20814, USA; erin.sheffels@gmail.com

**Keywords:** RAS, KRAS, HRAS, NRAS, SOS1, SOS2, RTK, SHP2, resistance

## Abstract

The *RAS* family of oncogenes (*HRAS*, *NRAS*, and *KRAS*) are among the most frequently mutated protein families in cancers. *RAS*-mutated tumors were originally thought to proliferate independently of upstream signaling inputs, but we now know that non-mutated wild-type (WT) RAS proteins play an important role in modulating downstream effector signaling and driving therapeutic resistance in *RAS*-mutated cancers. This modulation is complex as different WT RAS family members have opposing functions. The protein product of the *WT RAS* allele of the same isoform as mutated *RAS* is often tumor-suppressive and lost during tumor progression. In contrast, RTK-dependent activation of the WT RAS proteins from the two non-mutated WT *RAS* family members is tumor-promoting. Further, rebound activation of RTK–WT RAS signaling underlies therapeutic resistance to targeted therapeutics in *RAS*-mutated cancers. The contributions of WT RAS to proliferation and transformation in *RAS*-mutated cancer cells places renewed interest in upstream signaling molecules, including the phosphatase/adaptor SHP2 and the RasGEFs SOS1 and SOS2, as potential therapeutic targets in *RAS*-mutated cancers.

## 1. Introduction

**Figure 1:**
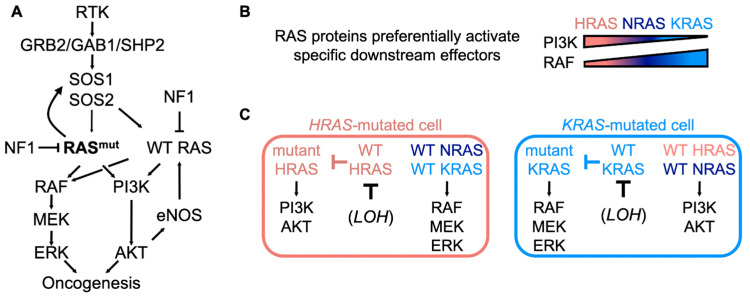
Mutant RAS and WT RAS cooperate to promote oncogenesis. (**A**) Schematic showing mutant RAS and WT RAS signaling in *RAS*-mutated cancer cells. (**B**) RAS family members show differential activation of downstream RAS effectors. HRAS activates PI3K well but RAF relatively poorly; KRAS activates RAF well but PI3K poorly. (**C**) Schematic showing proposed activation of RAF/MEK/ERK versus PI3K/AKT signaling in *HRAS*- and *KRAS*-mutated cancer cells. In *HRAS*-mutated cells, mutant HRAS activates PI3K/AKT signaling, whereas RTK-WT N/KRAS activate RAF/MEK/ERK signaling. WT HRAS is tumor suppressive and inhibits mutant HRAS signaling. In *KRAS*-mutated cells, mutant KRAS activates RAF/MEK/ERK signaling, whereas RTK-WT H/NRAS activate PI3K/AKT signaling. WT KRAS is tumor suppressive and inhibits mutant KRAS signaling.

The RTK/RAS pathway (Figure 1A) is among the most commonly mutated pathways in cancer [1,2]. The three *RAS* genes, *HRAS*, *NRAS*, and *KRAS*, encode four highly homologous protein isoforms (HRAS, NRAS, KRAS4A, and KRAS4B), driver mutations in *RAS* genes occur in ~20% of human tumors (reviewed in [3]). *KRAS* is the most frequently mutated *RAS* family member (75% of *RAS* mutations), including high incidence of mutations in lung [4], colon [5], and pancreatic cancers [6], three of the top four causes of cancer-related death [2,7,8]. *HRAS* and *NRAS* mutations are common in other cancer types including head and neck, skin, and hematopoietic cancers [9]. *RAS*-mutated cancers respond poorly to standard chemotherapy [10,11,12,13,14], so targeted approaches are needed to treat patients with *RAS*-mutated tumors. While advances in targeting specific mutant RAS proteins have been made [15,16,17,18], the majority of *RAS*-mutated tumors remain resistant to currently available treatments [4,12,14,19,20]. Novel strategies for targeting the RAS pathway are necessary to provide effective therapeutics to the majority of patients with *RAS*-mutated cancers. Understanding the signaling context of mutant RAS is key to developing indirect targeting and combination therapy strategies to better manage these cancers.

While typical models of oncogene activation assume that the mutated protein drives oncogenesis separately from the wild-type family members, the evidence that non-mutant wild-type (WT) RAS proteins influence cancer initiation and growth in *RAS*-mutated cancers is now well-established and several mechanisms for the effects have been proposed. Here, we summarize the current understanding of the effects of WT RAS on *RAS*-mutated cancers and the proposed mechanisms behind those effects.

## 2. Contributions of WT RAS to Mutant RAS-Driven Cancers

Non-mutant WT RAS proteins play an important role in modulating downstream effector signaling and oncogenesis in *RAS*-mutated cancers. While the contributions of WT RAS to *RAS*-mutated cancers varies based on factors such as the specific RAS isoform that is mutated and the cancer type, WT RAS proteins can be broadly categorized into two groups with opposing biologic functions. The protein product of the WT *RAS* allele of the same isoform as mutated *RAS* is tumor-suppressive, whereas the WT RAS proteins from the two non-mutated WT RAS family members are tumor-promoting (Figure 1B and reviewed in [21]).

### 2.1. The WT RAS Allele of the Same Isoform as Mutated RAS Inhibits Tumorigenesis

Several studies have found that the WT allele corresponding to the specific mutated *RAS* gene (WT *HRAS* in *HRAS*-mutated cancers [22,23]; WT *NRAS* in *NRAS*-mutated cancers [24,25]; WT *KRAS* in *KRAS*-mutated cancers [23,26,27,28,29,30]) suppresses tumorigenesis. Further, many *RAS*-mutated cancers have loss of heterozygosity (LOH) at the mutated gene, suggesting that loss of the wild-type allele confers a growth advantage. Evidence for LOH as a frequent event in cancer initiation has been observed in model systems for all three *RAS* genes (*HRAS* [23,31,32,33,34], *NRAS* [21,24,35], *KRAS* [21,36,37,38,39]). A survey of human tumor samples, cancer cell lines, and xenografts of lung, pancreatic, and colorectal cancers found mutant allele specific imbalance (MASI), where the mutant allele makes up more than half of the gene copies, in 58% of *KRAS*-mutated samples; over half of these imbalances were due to complete loss of the wild-type allele (uniparental disomy) [40]. Other surveys of both *KRAS*-mutated [41,42] and *HRAS*-mutated [43] patient tumor samples have found similar results. *KRAS* MASI is associated with worse prognosis in colorectal cancer and pancreatic cancer [44] and loss of the WT *KRAS* allele has been found at a higher rate in metastatic *KRAS*-mutated lung and pancreatic cancers compared to the primary tumors [36,37]. Several mechanisms have been proposed for inhibition of mutant RAS by the corresponding wild-type RAS. MASI and concomitant loss of the wild-type allele would increase the dosage of the mutant allele, increasing the number of mutant RAS proteins signaling in the cell, potentially increasing the oncogenic growth signal. Increased copy number of *KRAS* has been correlated with increased cell fitness in AML and CRC cells [41] and more aggressive and undifferentiated states in metastatic murine PDAC cells [45]. In patients, *KRAS* mutations combined with copy number gains were associated with decreased survival in lung cancer compared to *KRAS* mutations without copy number gains (LOH due to uniparental disomy or no LOH) [40].

Increased dosage of the mutant allele due to MASI does not, however, fully account for the effects of WT RAS of the same isoform; the level of WT KRAS also plays an important role in determining the extent of mutant KRAS-driven tumorigenesis. To examine the effects of WT KRAS levels on mutant KRAS-driven tumorigenesis, To et al. [27] crossed the *Kras2*^LA2^ lung cancer model into different mouse strains that show differing amounts of expression from the endogenous WT *Kras* allele. They found an inverse correlation between WT KRAS expression and tumorgenesis; mouse strains with lower relative expression of the WT *Kras* allele showed enhanced tumorigenicity. These data indicated that changes in copy number of the mutant *KRAS* allele do not fully explain the impact of WT KRAS on inhibiting tumorigenesis. To further probe the effects of WT KRAS on mutant KRAS-driven tumorigenesis, Ambrogio et al. [29] used both mice and RASless MEFs containing floxed WT *Kras*, and found that removal of WT KRAS enhanced mutant KRAS-driven signaling, proliferation, and tumorigenesis. Mechanistically, they found that the ability of WT KRAS to heterodimerize with mutant KRAS was necessary for its inhibitory function, as a dimerization deficient WT KRAS construct was unable to inhibit tumorigenesis. In contrast, they found that homodimers of mutant KRAS were essential for KRAS oncogenic function. These data corroborated findings that mutant KRAS may act as a dimer, requiring both monomers to be activated to achieve full downstream effector engagement [46,47]. In a cell with both constitutively active mutant KRAS and regulated WT KRAS, only dimers composed of two mutant KRAS proteins would provide a full oncogenic signal. Loss of WT KRAS, either through MASI or decreased expression of the wild-type allele, would increase the fraction of dimers composed of two mutants, increasing the oncogenic signal output. In addition to KRAS, both HRAS and NRAS have all been reported to dimerize [29,46,47,48,49], but the biologic effects of RAS dimerization and whether a similar mechanism underlies the tumor suppressive functions of WT HRAS and NRAS are untested. Of the three RAS family members, inhibition of mutant KRAS by WT KRAS is the most consistently observed across cancer types. KRAS dimerization may be the primary contributor, although changes in dosage of the mutant protein due to loss of the wild-type allele likely also impact the oncogenic signal. In cancers with copies of the wild-type allele remaining, the inhibition of mutant RAS by the corresponding wild-type RAS isoform indicates that therapies that specifically target the oncogenic mutant may perform better than therapies that target the mutant and wild-type proteins [36].

### 2.2. WT RAS Family Members Distinct from the Mutated RAS Allele Promote Oncogenesis

While WT RAS of the same isoform generally inhibits tumor initiation and growth, the protein products of the two non-mutated, WT *RAS* genes (for example *HRAS* and *NRAS* in a *KRAS*-mutated cancer; hereafter called WT *RAS* in all cases) are tumor promoting in RAS-mutated tumors [30,50]. Deletion of WT *HRAS* lead to decreased proliferation and increased apoptosis in *KRAS*-mutated endometrial cancer cells [30]. Knockdown of both WT *RAS* family members decreased proliferation in cell lines with mutated *HRAS* (T24), *NRAS* (RD), and *KRAS* (Mia PaCa 2) [50]. Mechanistically, knockdown of either the mutated *RAS* isoform or WT *RAS* differentially altered basal versus RTK-stimulated effector pathway activation [50]. Similar to signaling effects seen with oncogene-targeted inhibitors (see Section 4), these two signals cross-regulated each other: knockdown of either mutated *RAS* or WT *RAS* enhanced signaling through the other RAS pathway and simultaneous inhibition of both mutant RAS and WT RAS were required to promote apoptosis [50]. 

**Figure 2:**
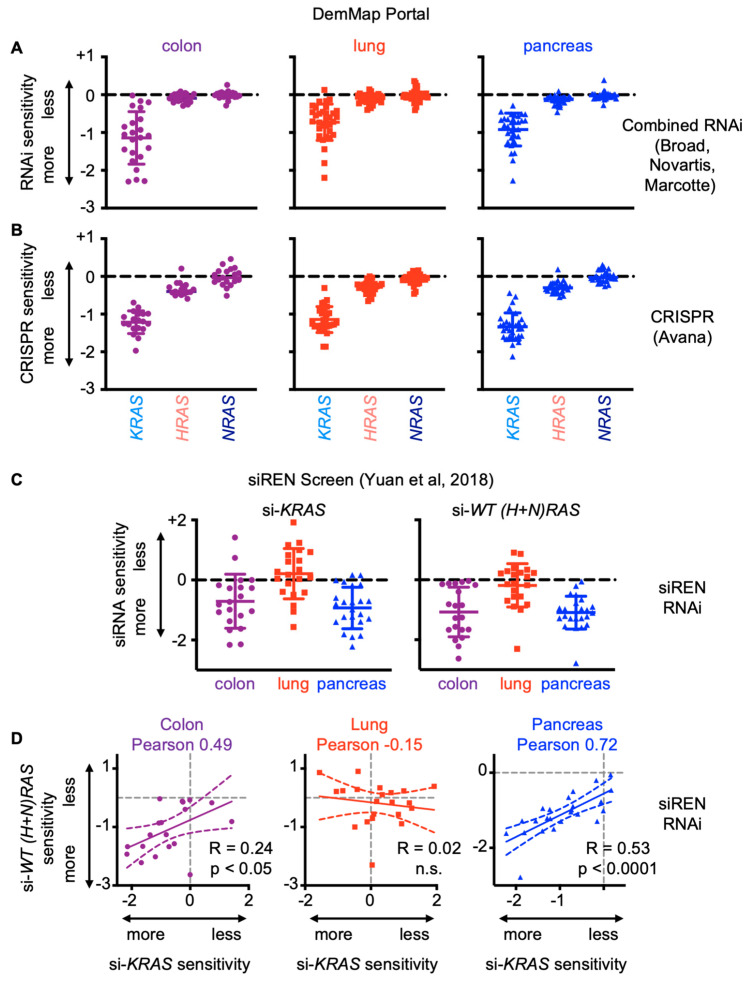
WT *HRAS*+*NRAS* knockdown correlates with mutant *KRAS* knockdown in *KRAS*-mutated colorectal and pancreatic cancer cell lines. (**A**,**B**) Gene effect of combined RNAi knockdown (Broad, Novartis, Marcotte, **A**) or CRISPR-mediated deletion (Avana, **B**) of *KRAS*, *HRAS*, or *NRAS* from the DepMap Portal data set in *KRAS*-mutated colorectal, NSCLC, and pancreatic cancer cell lines. (**C**,**D**) Effect of siRNA knockdown of mutant *KRAS* or both non-mutated WT *RAS* (*HRAS* and *NRAS*) genes (**C**) or linear correlation between mutant *KRAS* and WT *RAS* knockdown (**D**) from the siREN screen [23] in *KRAS*-mutated colorectal, NSCLC, and pancreatic cancer cell lines. Pearson correlation coefficient is shown. Each symbol indicates an individual cell line.

In in vivo models, deletion of individual WT *Ras* has shown variable effects on mutant RAS-driven tumorigenesis. In skin, WT *Kras* deletion promoted the progression of *Hras*-mutated papillomas to invasive squamous carcinomas, whereas WT *Nras* deletion decreased the formation of *Hras*-mutated papillomas [23]. Further, WT *Nras* deletion enhanced whereas WT *Hras* deletion inhibited *Kras*-mutated lung carcinogenesis [23]. In *KRAS*-mutated endometrial cancer cells, individual deletion of WT *HRAS* or *NRAS* limited proliferation in cancer cells, but not xenograft tumor growth [30]. These data suggest that the roles of WT RAS isoforms are complex and dependent on specific cellular context. To broadly examine the effects of WT *RAS* deletion in *KRAS*-mutated cancer cells, we performed a meta-analysis of mutated *KRAS*, WT *HRAS*, and WT *NRAS* dependencies in *KRAS*-mutated colorectal cancer, non-small cell lung cancer, and pancreatic cancer cell lines from both the Dependency Map Portal (DepMap) [51,52,53] and a large-scale siRNA knockdown screen that assessed RNAi depletion of RAS pathway ‘nodes’ (siRNEN Screen [54]). Analysis of both combined RNAi (Figure 2A) and AVANA/CRISPR screens (Figure 2B) showed a significant growth disadvantage in cells with mutant *KRAS* knockdown or deletion, but not in cells where *WT HRAS* or *NRAS* were individually removed. In contrast to DepMap data, which analyzed individual gene knockdowns, the siREN screen knocked down all genes of genetic ‘nodes’ simultaneously; thus the WT RAS node knocked down both *HRAS* and *NRAS*. In both colorectal cancer and pancreatic cancer cells, simultaneous knockdown of WT *HRAS* + *NRAS* limited survival to a similar extent as knockdown of mutated *KRAS* did (Figure 2C). Further, there was a direct correlation between individual cell line’s sensitivities to knockdown of mutated *KRAS* and WT *(H+N) RAS*, suggesting that mutant *KRAS* and WT *(H + N) RAS* act together to drive proliferation in these cells [54] (Figure 2D). Interestingly, the lung adenocarcinoma cells did not show dependency to either mutated *KRAS* or *WT RAS* in siREN screen data. This observation reflects previous observations that *KRAS*-mutated lung adenocarcinoma cells are “KRAS-independent” in 2D culture [55,56,57,58,59], but still require KRAS for anchorage-independent growth [60,61,62,63], and some *KRAS^G12C^*-mutated NSCLC cell lines respond to KRAS^G12C^ inhibitors in 3D culture and in vivo but not in 2D adherent culture [16]. Overall, these data highlight the importance of WT RAS signaling to promote tumorigenesis in *RAS*-mutated cancers.

## 3. Mechanisms of WT RAS Activation in *RAS*-Mutated Cancers

Multiple mechanisms have been proposed to describe the activation of WT RAS in the context of mutant RAS. Broadly, WT RAS activation has been described as either mutant RAS-dependent or RTK-dependent, although these two mechanisms are likely interdependent and act in concert to fully activate WT RAS.

### 3.1. Mutant RAS-Dependent and RTK-Dependent Mechanisms Activate WT RAS in RAS-Mutated Tumor Cells

The Kuriyan and Bar Sagi groups identified an allosteric RAS-GTP binding pocket on SOS1 that is distinct from the catalytic SOS1 domain [64]. RAS-GTP binding to this allosteric pocket relieves SOS1 autoinhibition [65,66], increasing SOS1 catalytic activity 80- to 500-fold [64] and activating a RAS-GTP–SOS1–WT RAS positive feedback that has been proposed to allow for ‘switch-like’ digital RAS activation [67,68,69]. This ‘switch-like’ behavior is important for normal childhood development [70,71], T and B cell development [72,73,74] and activation [75,76], and mutant KRAS-dependent cell proliferation and oncogenesis [77]. While SOS2 contains a homologous allosteric RAS-GTP binding site, whether SOS2 can be allosterically activated remains unconfirmed.

Independent of mutant RAS, RTK-dependent activation of WT RAS promotes activation of downstream effectors in parallel with constitutive mutant RAS signaling [50]. Knockdown studies showed that the non-mutated WT *RAS* genes are necessary for growth-factor-mediated signaling to RAS effector pathways in *HRAS*-, *NRAS*, and *KRAS*-mutated cancer cells [30,50], indicating that cancer cell response to growth factors may be mediated by WT RAS, not the oncogenic RAS mutant. RTK−WT RAS signaling supplements basal mutant RAS signaling to fully activate RAF/MEK/ERK and PI3K/AKT effector pathways [50,78] to promote proliferation [50] and G2 checkpoint integrity [79] in *RAS*-mutated cancer cells. Intriguingly, the McCormick lab showed that mutant RAS and WT RAS signals cross-regulate each other; knockdown of mutated *RAS* enhances RTK−WT RAS signaling to downstream effectors and conversely knockdown of WT *RAS* enhances basal RAS effector activation [50]. Due to this cross-regulation, which is likely due to rebound signaling (see Section 4), they further showed that combined inhibition of both mutant RAS and WT RAS signaling was necessary to induce apoptosis in *RAS*-mutated cancer cells.

These two mechanisms of WT RAS activation are not mutually exclusive and likely cooperate in some contexts. For example, positive feedback activation of SOS1 by active RAS-GTP potentiates EGF signaling to downstream effectors in vitro [66] and supports prolonged RAS and ERK activation downstream of T cell and B cell receptors [75,76]. Further, other signaling mechanisms can contribute to WT RAS activation in a context-specific manner. For example, endothelial nitric oxide synthase (eNOS) is phosphorylated and activated by RAS-AKT signaling; eNOS can in turn nitrosylate and activate WT HRAS generating a positive feedback loop that contributes to cellular transformation and tumor maintenance [80].

### 3.2. The RasGEFs SOS1 and SOS2 May Play Non-Overlapping Roles in Cells Expressing Oncogenic RAS

Data from our lab and others suggests that SOS1 and SOS2 may play non-overlapping roles in *RAS*-mutated tumors. Mutant KRAS–SOS1–WT RAS allosteric signaling promotes growth of *KRAS*-mutated pancreatic cancer cell xenografts [77], but has not been assessed for mutant HRAS- or NRAS-dependent transformation. In contrast, we found that RTK-SOS2-WT RAS signaling, but not allosteric SOS2 activation, is a critical mediator of mutant KRAS-driven transformation [81] by protecting *KRAS*-mutated cancer cells from anoikis [82]. We further showed that there was a hierarchical requirement for SOS2 to drive mutant RAS-dependent transformation, with KRAS > NRAS > HRAS. These data suggest that signaling via SOS1 and SOS2 may promote unique aspects of WT RAS signaling in *RAS*-mutated tumors.

### 3.3. WT RAS Cooperates with Mutant RAS to Fully Activate Downstream RAS Effector Pathways

Although the RAS isoforms are highly similar in terms of sequence and structure, the differences in their developmental requirement and mutation rates between cancer types indicate that they are not biologically equivalent. RAS isoforms have differing abilities to activate their downstream effectors [83,84] which are not correlated with a difference in binding affinity [85] or isoform stability [86]. Specifically, HRAS, NRAS, and KRAS show inverse abilities to activate PI3K/AKT signaling and RAF/MEK/ERK signaling: mutant HRAS is a potent activator of PI3K but a relatively poor activator of RAF; conversely, KRAS potently activates RAF but poorly activates PI3K [78,83,84], and NRAS shows intermediate activation of both RAF and PI3K effector pathways (Figure 1C). A recent study has shed light on the mechanism for the differential activation of RAF proteins [87]. Upon RAS activation, RAF proteins form homo- and heterodimers, with BRAF/CRAF heterodimers being the dominant complex responsible for downstream MEK/ERK activation to promote mutant KRAS-driven transformation [88]. BRAF preferentially interacts with KRAS via an interaction between the KRAS (4B) polybasic region and an acidic N-terminal region in BRAF [87]. The ability to directly associate with both BRAF and CRAF makes KRAS a more potent activator of the RAF/MEK/ERK cascade. While the precise mechanism for differential PI3K activation between HRAS and KRAS remains unclear, a major contributor seems to be the polybasic stretch in the hypervariable region (HVR) of KRAS; mutating basic residues in the KRAS (4B) HVR inhibits RAF/MEK/ERK signaling but enhances PI3K/AKT phosphorylation [89].

This differential effector activation by RAS isoforms leads to the proposed model that WT RAS contributes to cancer by signaling to effectors that the mutant RAS isoform cannot activate effectively [90]. *HRAS*-mutated cancer cells require RTK–WT RAS signaling to activate RAF/MEK/ERK signaling [78,91]. Conversely, in *KRAS*-mutated colorectal [92] and lung [93] adenocarcinoma cells, PI3K/AKT pathway activation is dependent on RTK signaling. Furthermore, we showed that in *KRAS*-mutated cancer cells, RTK-SOS2-WT RAS signaling was necessary to provide adequate PI3K/AKT signaling for cells to survive in anchorage-independent growth conditions (protection from anoikis) [81], but *HRAS*- and *NRAS*-mutated cancer cells could survive in anchorage-independent conditions without RTK-SOS2 supplemented PI3K signaling [82]. These different requirements for WT RAS isoforms in *RAS*-mutated cancers are also reflected in mutational activation of RAS and downstream RAS effectors. Analysis of co-mutation frequencies shows that *KRAS* and *BRAF* mutations are generally mutually exclusive, while *KRAS* and *PIK3CA* (the gene encoding the catalytic p110α subunit of PI3K) mutations co-occur frequently, consistent with the idea that KRAS already highly activates the RAF pathway, but requires supplemental signaling in the PI3K/AKT pathway [94,95]. Thus, WT RAS proteins may contribute to RAS effector activation in *RAS*-mutated cancer cells through their ability to activate the pathway(s) that the mutant RAS does not activate well (Figure 1).

## 4. WT RAS Signaling Underlies Resistance to Targeted Therapies in *RAS*-Mutated Cancers

### 4.1. Inhibitors of RAS Effector Pathways

Initial efforts to target *RAS*-mutated cancers focused on inhibiting downstream RAF/MEK/ERK and PI3K/AKT effector signaling, as RAS proteins have been historically difficult to target. Unfortunately, in multiple preclinical models of mutant RAS driven malignancies, single-agent MEK inhibitor treatment is ineffective. In *KRAS*-mutated cancer cells, single agent MEK inhibitor treatment is ineffective because it both relieves ERK-dependent negative feedback signaling and induces the expression of RTK and ligands [96,97,98,99,100,101,102,103,104,105]. These effects cause rapid RTK–WT RAS-dependent activation of both parallel PI3K/AKT signaling and the inhibited RAF/MEK/ERK cascade to drive therapeutic resistance. Similar rebound signaling occurs after MEK inhibitor treatment in *HRAS*- and *NRAS*-mutated cancer cells.

Similar to what is seen after single-agent MEK inhibitor treatment, rebound RTK signaling occurs after PI3K inhibition, with both RAF/MEK/ERK and PI3K/AKT rebound activation [106,107,108]. Combinations of MEK and PI3K/mTOR inhibitors were successful preclinically [98,109], but clinical success has been limited by toxicity [108,110,111]. Unfortunately, toxicity is likely unavoidable with this treatment strategy, since the RAF/MEK/ERK and PI3K/AKT pathways are both key players in normal cell function. To avoid this toxicity, many studies have investigated the efficacy of blocking the PI3K pathway indirectly, or finding other pathways that synergize with MEK or PI3K inhibition [92,112]. In *KRAS*-mutated colorectal [92] and lung [93] adenocarcinoma cells, PI3K/AKT pathway activation is dependent on RTK–WT RAS signaling (see Section 3.3), and thus inhibition of RTK signaling should indirectly inhibit PI3K activation. For example, Ebi et al. [92] showed that PI3K signaling was most often downstream of IGF-1R in *KRAS-*mutated colorectal cancer cells and that IGF-1R inhibition did indirectly block PI3K signaling and cooperate with MEK inhibitors to induce cell death.

**Figure 3:**
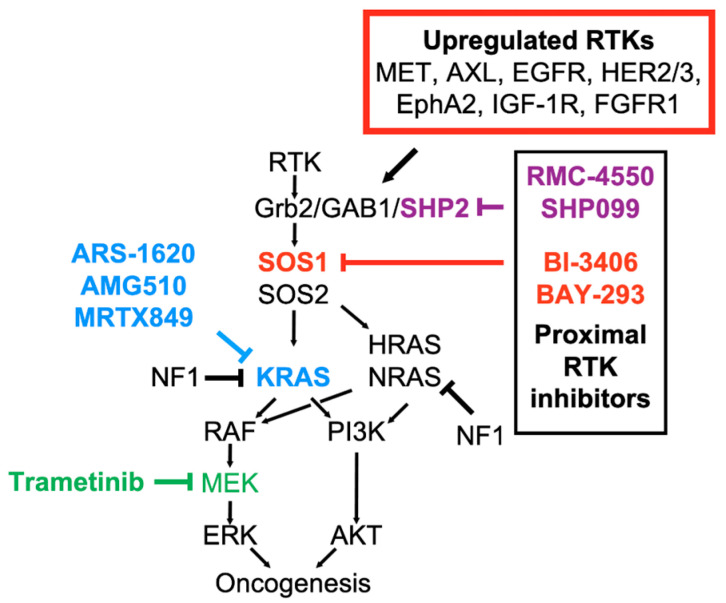
In *KRAS*-mutated cancer cells, treatment with either KRAS^G12C^ inhibitors or MEK inhibitors causes rebound RTK signaling leading to therapeutic resistance. Inhibition of proximal RTK signaling using inhibitors of the common proximal RTK signaling intermediates SHP2 or SOS1 can potentially limit resistance to oncogene-targeted therapies, thereby significantly prolonging the initial window of therapeutic efficacy.

Unfortunately, it is often difficult to identify which RTK must be co-inhibited with either MEK or PI3K inhibitors for a given tumor type. RNA-sequencing studies showed that MEK inhibitor treatment induces simultaneous upregulation of multiple RTKs and ligands [113,114], so co-inhibition of individual RTKs will likely be ineffective in blocking MEK inhibitor resistance [97,104,115]. Further, even specific experiments where a dominant RTK drove MEK inhibitor resistance, the specific RTK involved was either tumor type or more often cell line specific. IGF-IR, MET, ERBB1/2, ERBB3, PDGFRa, AXL and FGFR1 have all been implicated in MEK-inhibitor resistance in *KRAS*-mutated tumors depending on the anatomical tumor type or specific cell line that was examined [92,97,104,105] (Figure 3). These studies indicate that broad inhibition of proximal RTK signaling will likely be required to block MEK inhibitor resistance.

### 4.2. Mutant RAS Inhibition

While most early efforts to target RAS have been unsuccessful, recent breakthroughs in both our understanding of an older ‘unsuccessful’ RAS inhibitor and novel insights into the accessibility of specific RAS mutations have led to renewed hope for successful targeting of mutant RAS in the clinic. 

#### 4.2.1. Tipifarnib as an HRAS-Specific Inhibitor

For mutant HRAS, advances in our understanding of the enzymes responsible for post-translational lipid modification of RAS isoforms have showed that farnesyltransferase inhibitors (FTIs), drugs originally designed as pan-RAS inhibitors, specifically inhibit HRAS and have clinical activity for patients with *HRAS*-mutated tumors. All RAS isoforms are post-translationally modified by the covalent addition of a C15 farnesyl isoprenoid lipid at their C-terminus and this modification is required for their membrane association and biological activity. This process, known as prenylation, is normally catalyzed by the enzyme farnesyltransferase (FTase). Since prenylation is required for RAS biological activity, FTIs were developed and tested as therapeutics in *RAS*-mutated cancers [12]. Unfortunately, KRAS and NRAS, the major RAS isoforms that are mutated in adult cancers, can be alternatively prenylated by gerangeranyltransferases [116,117,118], and FTIs failed in Phase III clinical trials for *KRAS*-mutated colorectal [119] and pancreatic [120] cancers. In contrast, HRAS is exclusively farnesylated [121] and its membrane association is inhibited by the FTI tipifarnib. In Phase II trials, patients with *HRAS*-mutated head and neck squamous cell carcinoma [122], urothelial carcinoma [123], and salivary gland cancer [124] have shown encouraging clinical responses to tipifarnib. Similar to MEK inhibitors (above) and KRAS^G12C^ inhibitors (below), tipifarnib treatment of *HRAS*-mutated cancer cells show adaptive reactivation RTK–WT RAS signaling and enhanced RAF/MEK/ERK pathway activation [91]. Furthermore, rebound RTK signaling after tipifarnib treatment occurred through multiple different RTKs in vivo [91], suggesting the need for novel therapeutic combinations.

#### 4.2.2. Covalent KRAS^G12C^ Inhibitors

Unlike other mutant KRAS proteins, the active -SH group on the cysteine of G12C-mutant KRAS allows for covalent modification using therapeutics. The Shokat lab combined this idea with their discovery of a novel binding pocket on KRAS that is only present in the GDP (inactive) state to develop the first KRAS^G12C^ inhibitor that binds KRAS in the GDP-bound (inactive) state and covalently modifies the mutant cysteine in KRAS^G12C^ [15]. Since this first report, there has been a flurry of novel compounds including the bioavailable tool compound ARS-1620 [16] and several clinical compounds, including AMG 510 [17] and MRTX849 [18], both of which are currently in clinical trials for *KRAS^G12C^*-mutated solid tumors. Preliminary reports of patient responses to these drugs are encouraging: ~50% of patients with *KRAS^G12C^*-mutated NSCLC show partial responses to either AMG 510 or MRTX849, and a majority of the remaining patients show disease stabilization [17,18]. Similar to both FTIs and MEK inhibitors, rapid resistance to KRAS^G12C^ inhibitors develops. In vitro and in vivo studies revealed upregulated RTK signaling [18,114,125,126] and potential synthesis of new uninhibited KRAS^G12C^ [126] as the major drivers of KRAS^G12C^ inhibitor resistance. Similar to MEK inhibitors, the specific RTK driving KRAS^G12C^ inhibitor resistance is cell type specific, so that while individual RTK inhibitors might be effective in blocking KRAS^G12C^-inhibitor resistance in a specific cancer cell line, broad inhibition of RTK signaling will be required to delay therapeutic resistance and make KRAS^G12C^ inhibitors clinically efficacious [18,125,126,127].

### 4.3. Inhibition of Proximal RTK Signaling Can Overcome MEK- and KRAS^G12C^-Inhibitor Resistance

Unbiased genetic and pharmacologic screens revealed three distinct classes of synthetic lethal targets that synergize with both MEK and KRAS^G12C^ inhibitors in *KRAS*-mutated cancer cells [112,127,128]: (i) individual RTKs or proximal RTK signaling components (including SHP2 and SOS1) whose inhibition can broadly inhibit RTK signaling, (ii) mTOR/PI3K survival signaling components, and (iii) regulators of cell cycle progression. Both mTOR/PI3K pathway inhibitors [128,129] and CDK4/6 inhibitors [127,130] potentiate the effects of MEK inhibitors and KRAS^G12C^ inhibitors in xenograft studies, suggesting that targeting these collateral dependencies may be a viable therapeutic strategy. Since PI3K activation is downstream of RTK–WT RAS signaling in *KRAS*-mutated cancer cells [92,93] and cell cycle progression requires RTK/RAS signaling, these collateral dependent targets may represent a common mechanism for inhibiting MEK- and KRAS^G12C^-inhibitor resistance [127]. Here, the discovery of potent, orally available SHP2 and SOS1 inhibitors has the potential to dramatically augment oncogene-targeted therapies for *RAS*-mutated cancer.

### 4.4. SOS1 and SHP2 Are Therapeutic Targets in RAS-Mutated Cancer Cells

SHP2 and SOS1 are common proximal RTK signaling intermediates; the development of potent, specific inhibitors for both SHP2 (SHP099 [131,132]; RMC-4550 [133]) and SOS1 (BAY-293 [134]; BI-3406 [135,136]) has led to new approaches to treating *RAS*-mutated cancers. Both SHP2 [131,132,133] and SOS1 [134,135,136] inhibitors are effective in inhibiting cell growth in situ as single agents in cells with RTK/RAS pathway mutations that are dependent upon RAS nucleotide cycling, including cells with *EGFR* mutations, *KRAS* (G12/13) mutations, LOF *NF1* mutations, and *BRAF* type III mutations, but not in cells with *KRAS* Q61 mutations, *BRAF* Type I/II mutations, or concomitant *PIK3CA* mutations. In xenograft studies using adult lung, pancreas, or colon cancer cell lines, SHP2 inhibitors enhanced the efficacy of covalent KRAS^G12C^ inhibitors [18,114] and both SHP2 [113,137] and SOS1 [135] inhibitors enhanced the efficacy of MEK inhibitors. Intriguingly, although neither SHP2 nor SOS1 inhibitors were able to inhibit cancer cells with *KRAS* Q61 mutations as single agents [133,135], both were able to enhance the efficacy of the MEK inhibitor trametinib in xenograft models harboring *KRAS* Q61 mutations [113,135], suggesting that inhibiting proximal RTK signaling might be broadly effective in combination therapies for *RAS*-mutated tumors harboring G12, G13, or Q61 mutations. In *HRAS*- or *NRAS*-mutated cells, neither SHP2 or SOS1 inhibitors are effective as single agents [133,135,138], however, a recent study showed that while *NRAS^Q61^*-mutated neuroblastoma cells were insensitive to SHP2 inhibitors alone, combined SHP2 and MEK inhibition showed synergistic inhibition of cell growth [138], suggesting that proximal RTK (SHP2 or SOS1) inhibitors may be a general therapeutic option to overcome MEK inhibitor resistance in *RAS*-mutated cancer cells.

### 4.5. The Spectrum of KRAS Mutations between Different Cancer Types Leads to Cancer-Specific Vulnerabilities to WT RAS Inhibition

*KRAS* is the most frequently mutated *RAS* gene; KRAS mutations occur in 32–35% of lung adenocarcinomas (LUAD), 41–50% of colorectal adenocarcinomas (COAD), and 86–88% of pancreatic adenocarcinomas (PAAD) [3,8]. While G12 mutations predominate each of these cancers, there are cancer-specific differences in the *KRAS* mutational spectrum that have functional consequences for therapeutics targeting WT RAS signaling [8].

In LUAD, 40% of *KRAS* mutations are G12C, whereas *KRAS^G12C^* mutations occur less frequently in COAD (7%) and PAAD (1%) [8,11,14]. Due to these mutational differences, covalent G12C inhibitors (see Section 4.2.2) will likely have the greatest impact in LUAD, where ~50% of patients have shown partial responses in Phase I and II trials [17,18]. Similar to MEK inhibition, treatment with covalent KRAS^G12C^ inhibitors causes rapid rebound activation of multiple RTKs, making RTK–SOS1/2–WT RAS signaling an important therapeutic target in *KRAS^G12C^*-mutated LUAD.

In late-stage colorectal adenocarcinoma, the monoclonal antibodies cetuximab and panitumumab, which inhibit the EGFR, improved outcomes for patients with *WT KRAS* but not with KRAS mutations [139,140,141,142,143]; these anti-EGFR therapies are FDA approved for first-line treatment for patients with *WT KRAS* colorectal cancers where they used in combination with conventional chemotherapy [144]. Intriguingly, retrospective analysis of the Phase III trial data assessing the efficacy of cetuximab in COAD showed that patients with *KRAS^G13D^* mutations may benefit from anti-EGFR therapies [145], although subsequent Phase II trials that prospectively assessed anti-EGFR therapies in patients with *KRAS^G13D^* mutations have shown varying results [146,147,148]. COADs have a high percentage of *KRAS^G13D^* mutations (17%) compared to either LUAD (3%) or PAAD (<1%) [8]. A recent manuscript by McFall et al. [149] has shed light on why colorectal cancers with *KRAS^G13D^* mutations might be sensitive to anti-EGFR therapies. In cells with *KRAS^G13D^* mutations, WT RAS activation is particularly sensitive to EGFR inhibition [149]. KRAS G12 mutant proteins interact strongly with the RasGAP NF1 and this strong interaction competitively inhibits NF1, activating wild-type HRAS and NRAS independent of EGFR. In contrast, mutant KRAS^G13D^ proteins have a relatively weak interaction with NF1, allowing NF1 to inactivate wild-type HRAS and NRAS in the absence of EGFR stimulation and making WT RAS signaling EGFR-dependent in these cells [149]. Due to this, downstream signaling in G13D-mutated cells is extremely RTK-dependent, possibly explaining why *KRAS^G13D^*-mutated colorectal cancers are sensitive to EGFR-TKIs while other *KRAS*-mutated colorectal tumors are refractory to EGFR-TKI treatment [145]. Rabara et al. [150] confirmed these results and further showed that a subset of *KRAS^G13D^*-mutated colorectal adenocarcinomas had co-mutation of *NF1*. Only *KRAS^G13D^*-mutated cancers with WT NF1 were responsive to EGFR inhibition.

In PAAD, 17% of *KRAS* mutations are G12R, whereas *KRAS^G12R^* mutations only occur in ~1% of COAD and LUAD [8]. Hobbs et al. [151] found that *KRAS^G12R^*-mutated PAAD cells have unique signaling properties that may make them vulnerable to WT RAS inhibition. Pancreatic cancer cells are dependent on RAS-driven macropinocytosis for nutrient uptake and survival [152,153]. Using a panel of *KRAS*-mutated PAAD cell lines, Hobbs et al. [150] showed that while macropinocytosis was KRAS-dependent in *KRAS^G12D^* and *KRAS^G12V^*-mutated cells, macropinocytosis was KRAS-independent in cells with *KRAS^G12R^* mutations. They found that compared to cells with G12D or G12V mutations, *KRAS^G12R^*-mutated cells showed defective PI3K-AKT signaling, due to the inability of KRAS^G12R^ to interact with the p110α catalytic subunit of PI3K. Macropinocytosis was PI3Kγ-dependent in *KRAS^G12R^*-mutated cells, suggesting that WT RAS signaling was specifically required for nutrient uptake in these cells. Due to these unique signaling properties, *KRAS^G12R^*-mutated cells were more sensitive to single-agent PI3K or MEK inhibition compared with *KRAS^G12D^* and *KRAS^G12V^*-mutated cells [151]. In addition to its inability to interact with p110α, KRAS^G12R^ cannot interact with the catalytic domain of SOS1 [151], and isogenic NCI-H23 cells expressing KRAS^G12R^ were insensitive to SOS1 inhibition [135].

To investigate these findings in a controlled model, Zafra et al. [154] recently generated an in vivo *Kras* allelic series where they directly compared tumorigenesis and drug sensitives of *Kras^G12C^*, *Kras^G12D^*, *Kras^G12R^*, and *Kras^G13D^* mutants. In keeping with clinical observations, G12C and G12D mutations showed overall enhanced tumorigenesis in both the colon and pancreas compared to G12R or G13D mutations. Further, when assessing drug sensitivities in pancreatic organoids, *Kras^G13D^*-mutated organoids were much more sensitive to EGFR inhibition alone compared with other mutants, and *Kras^G12C^*-mutated organoids were sensitive to combining an EGFR inhibitor with covalent KRASG12C inhibition [154], paralleling the findings described above. Taken together, these data indicate that specific *KRAS* mutations may be more sensitive to inhibitors of WT RAS signaling, leading to organ-specific vulnerabilities based on mutation frequencies.

## 5. Conclusions

WT RAS signaling is an important modifier of *RAS*-mutated oncogenesis, and inhibition of WT RAS signaling may be required for effective treatment of *RAS*-mutated cancers. Understanding the mechanisms by which WT RAS is activated is an important step in determining the best ways to limit WT RAS signaling. The ability to pharmacologically manipulate the common proximal signaling intermediates SHP2 and SOS1/2 may lead to optimized therapeutic combinations that can be used to treat *RAS*-mutated cancers.

## Data Availability

The data presented in this study are available in supplemental Table S1. Data were downloaded from https://www.depmap.org/depmap, accessed on 27 April 2021 (DepMap Data) and from [54] (siREN Screen Data).

## Supplementary Materials

The following are available online at https://www.mdpi.com/article/10.3390/genes12050662/s1.
